# Correction: Molecular adaptation in Rubisco: Discriminating between convergent evolution and positive selection using mechanistic and classical codon models

**DOI:** 10.1371/journal.pone.0196267

**Published:** 2018-04-18

**Authors:** Sahar Parto, Nicolas Lartillot

The Funding statement is incorrect. The correct statement is: Natural Sciences and Engineering Research Council of Canada (NSERC), ANR-15-CE32-0005 « Convergenomix ». Part of the analyses were run on the PRABI cluster.
