# Supplementary material for: Molecular adaptation in Rubisco: Discriminating between convergent evolution and positive selection using mechanistic and classical codon models
Source: PLoS One. 2018 Feb 12;13(2):e0192697. doi: 10.1371/journal.pone.0192697 (PMC5809049; doi:10.1371/journal.pone.0192697)
Supplement: S1 Fig — (DOCX) [file pone.0192697.s001.docx]

| 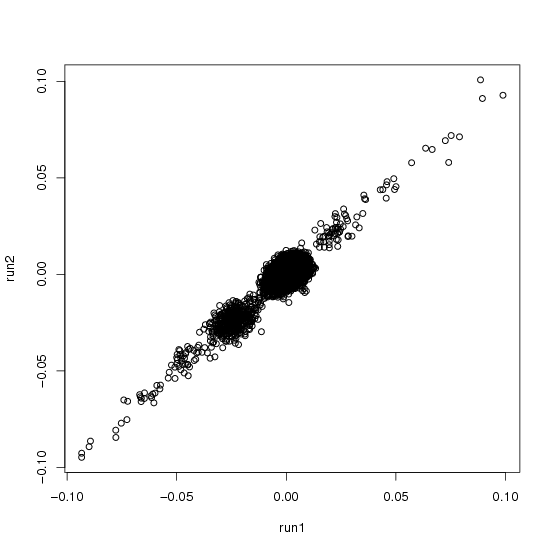  ***a*** |
| --- |
| 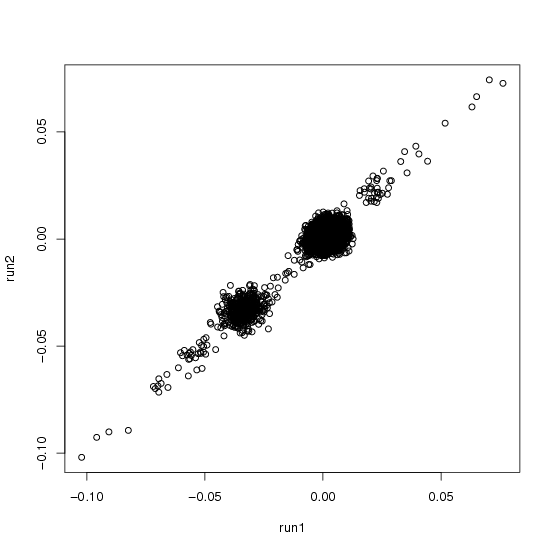  ***b*** |

Figure S 1. Estimates of posterior mean differential selection effects across all amino acids and all sites for two independent chains, for C3 plants (*a*) and C4 plants (*b*). The correlation coefficient is 0.78 and 0.81, respectively.
