# Supplementary material for: Molecular adaptation in Rubisco: Discriminating between convergent evolution and positive selection using mechanistic and classical codon models
Source: PLoS One. 2018 Feb 12;13(2):e0192697. doi: 10.1371/journal.pone.0192697 (PMC5809049; doi:10.1371/journal.pone.0192697)
Supplement: S2 Fig — (DOCX) [file pone.0192697.s002.docx]

| 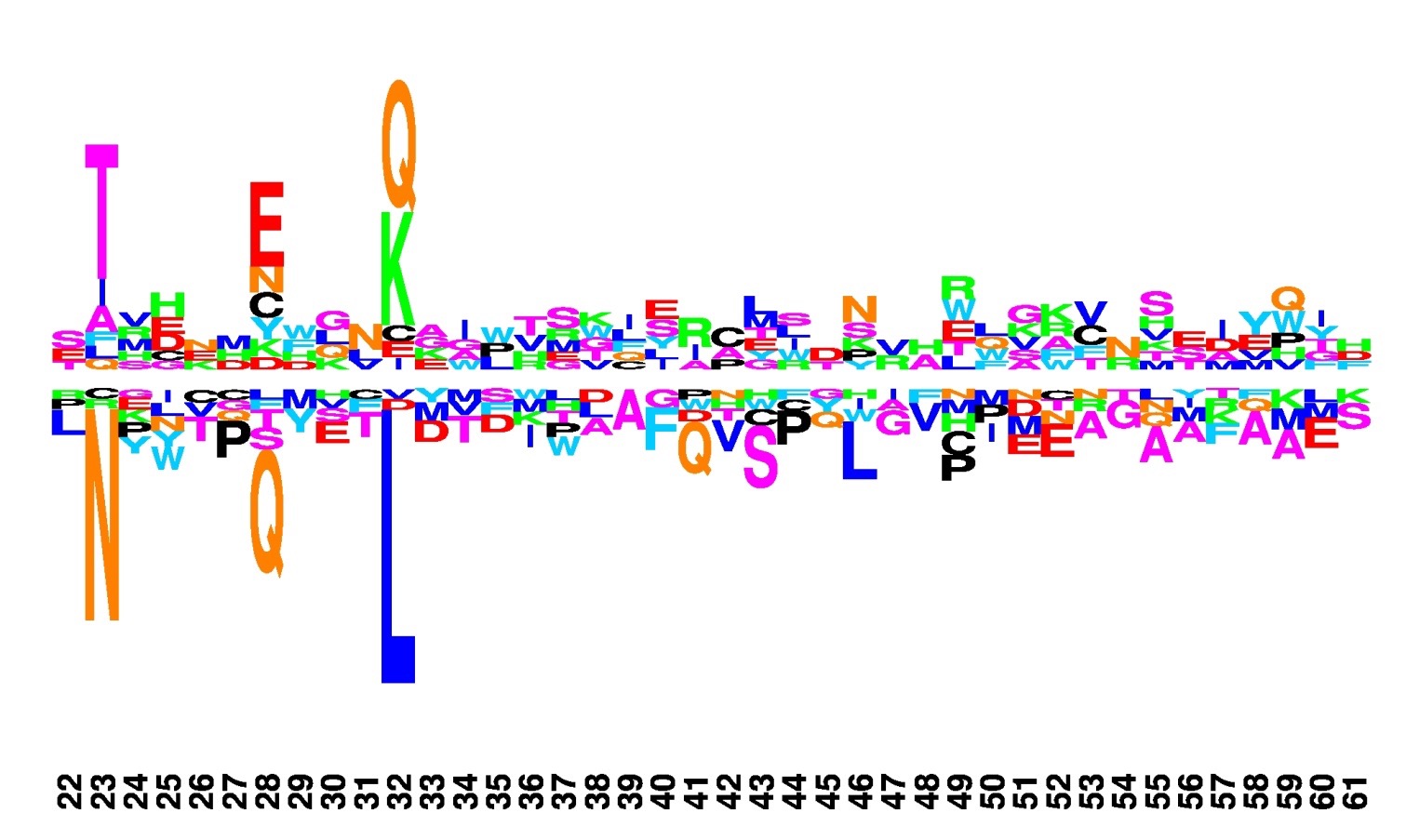 |
| --- |
| 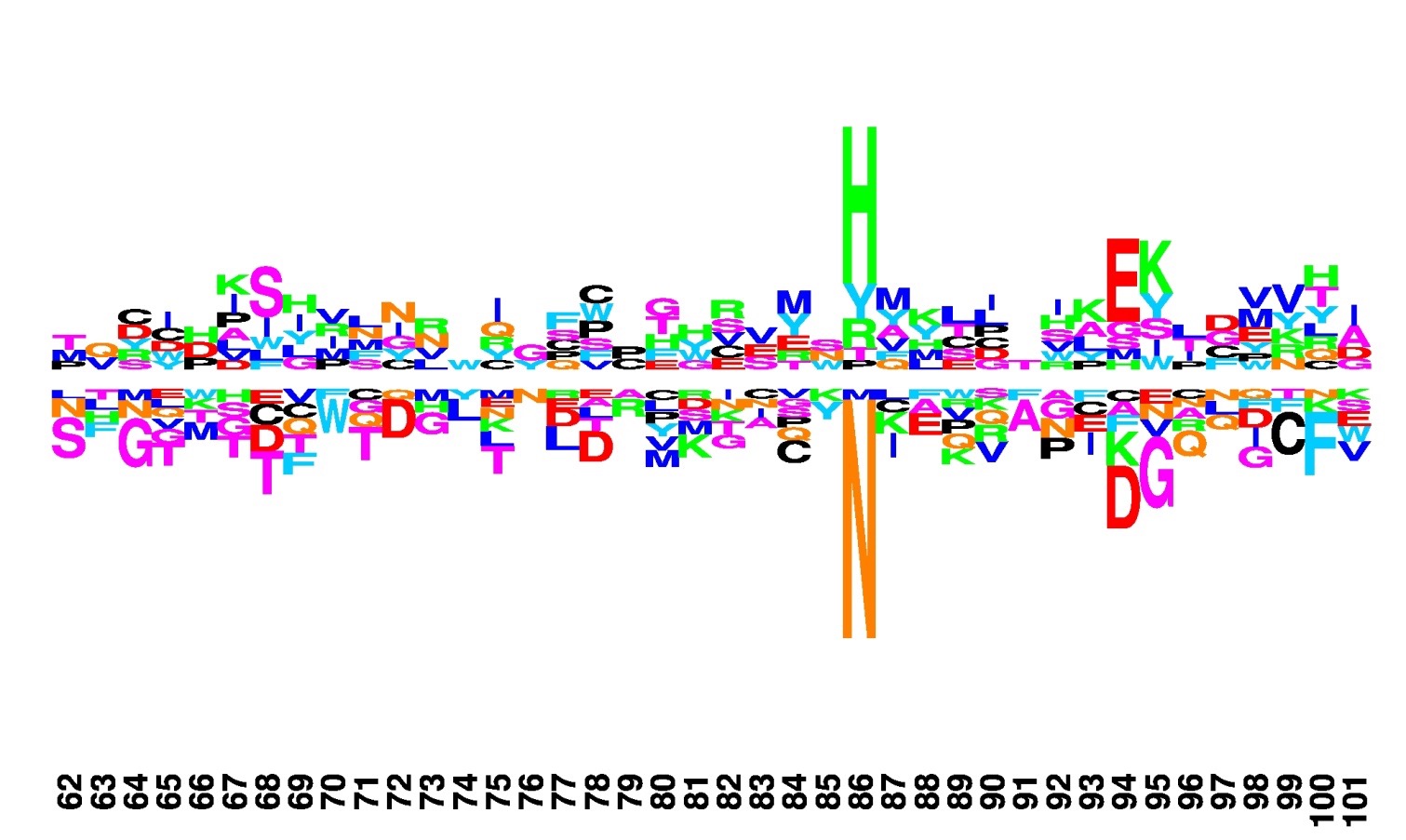 |
| 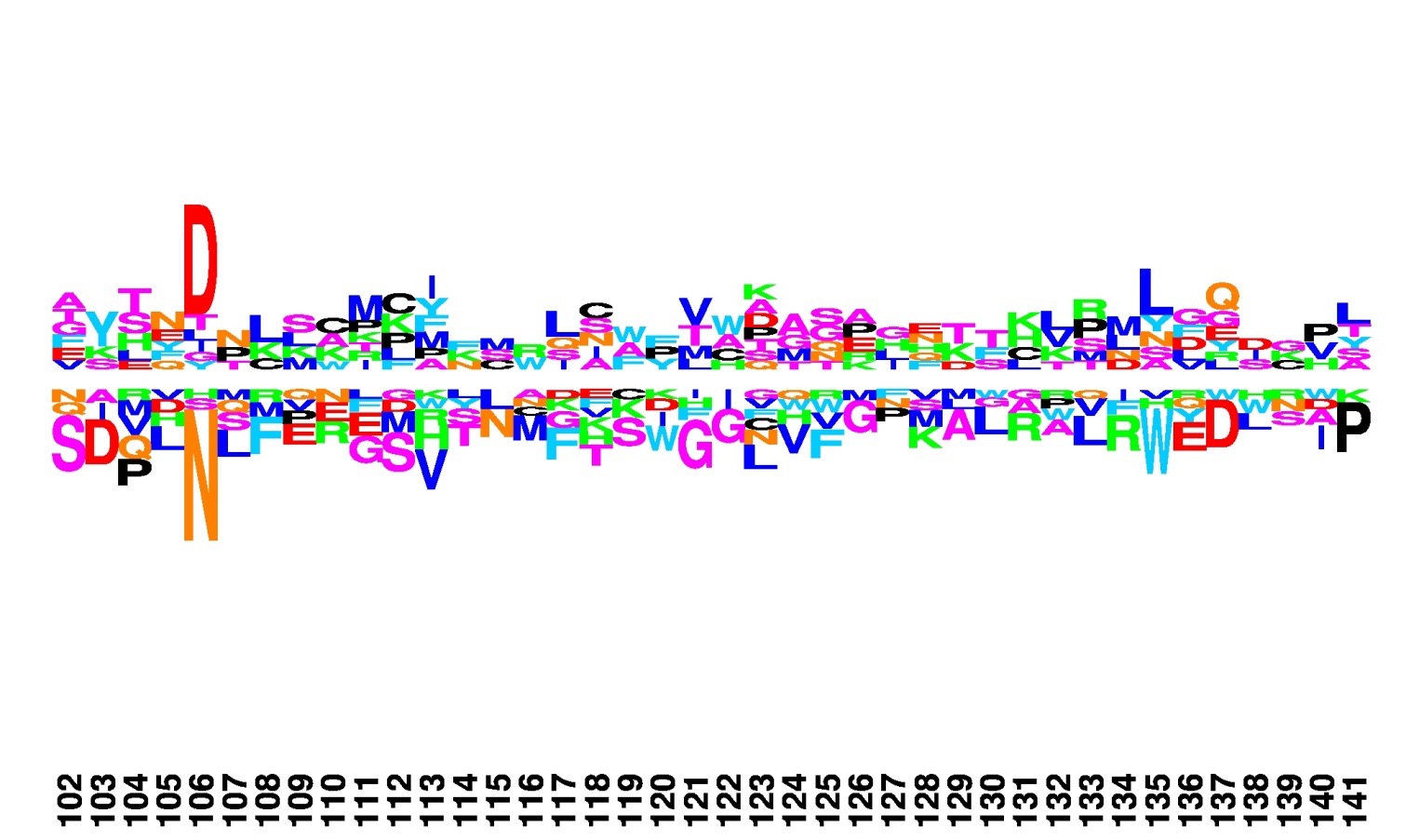 |
| 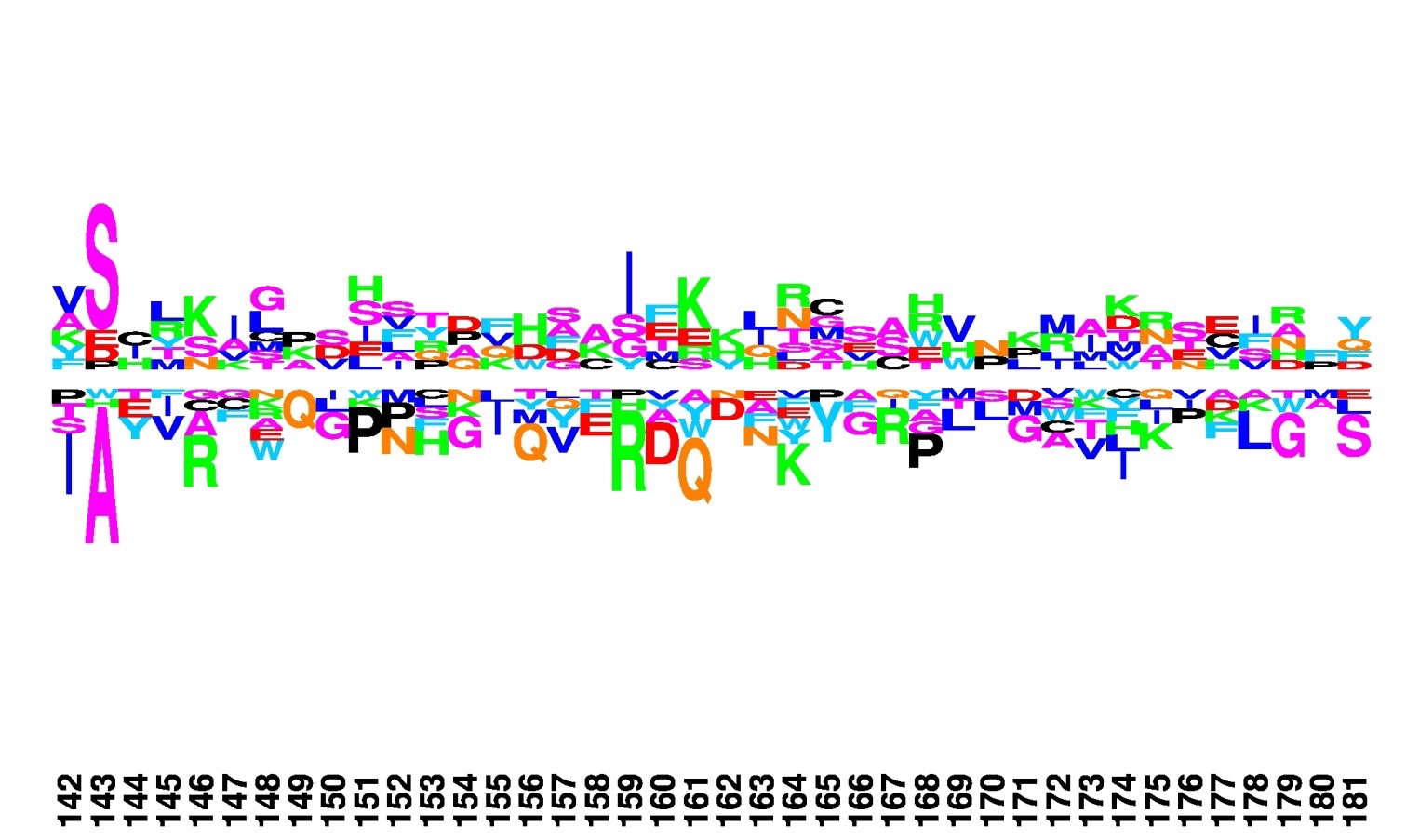 |
| 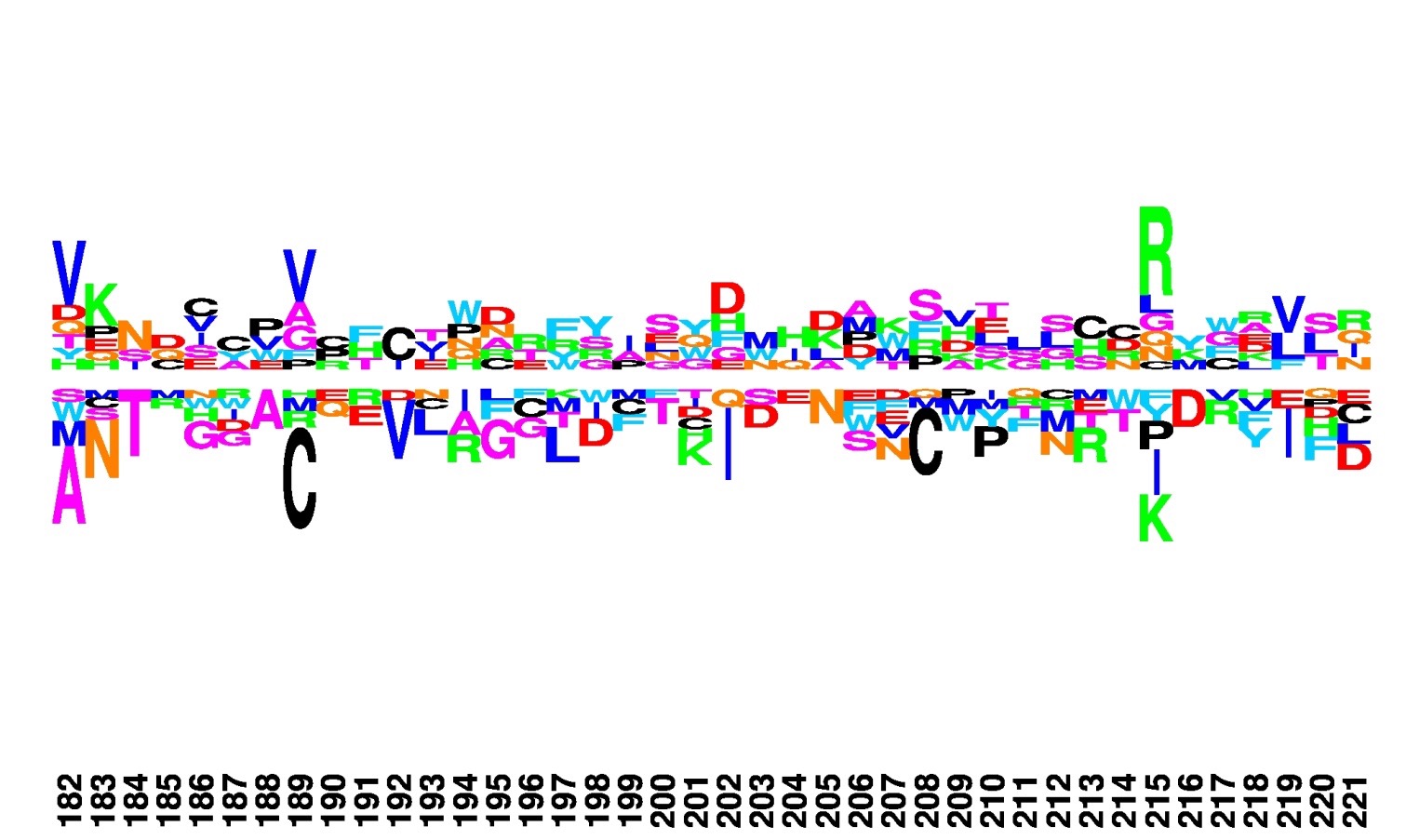 |
| 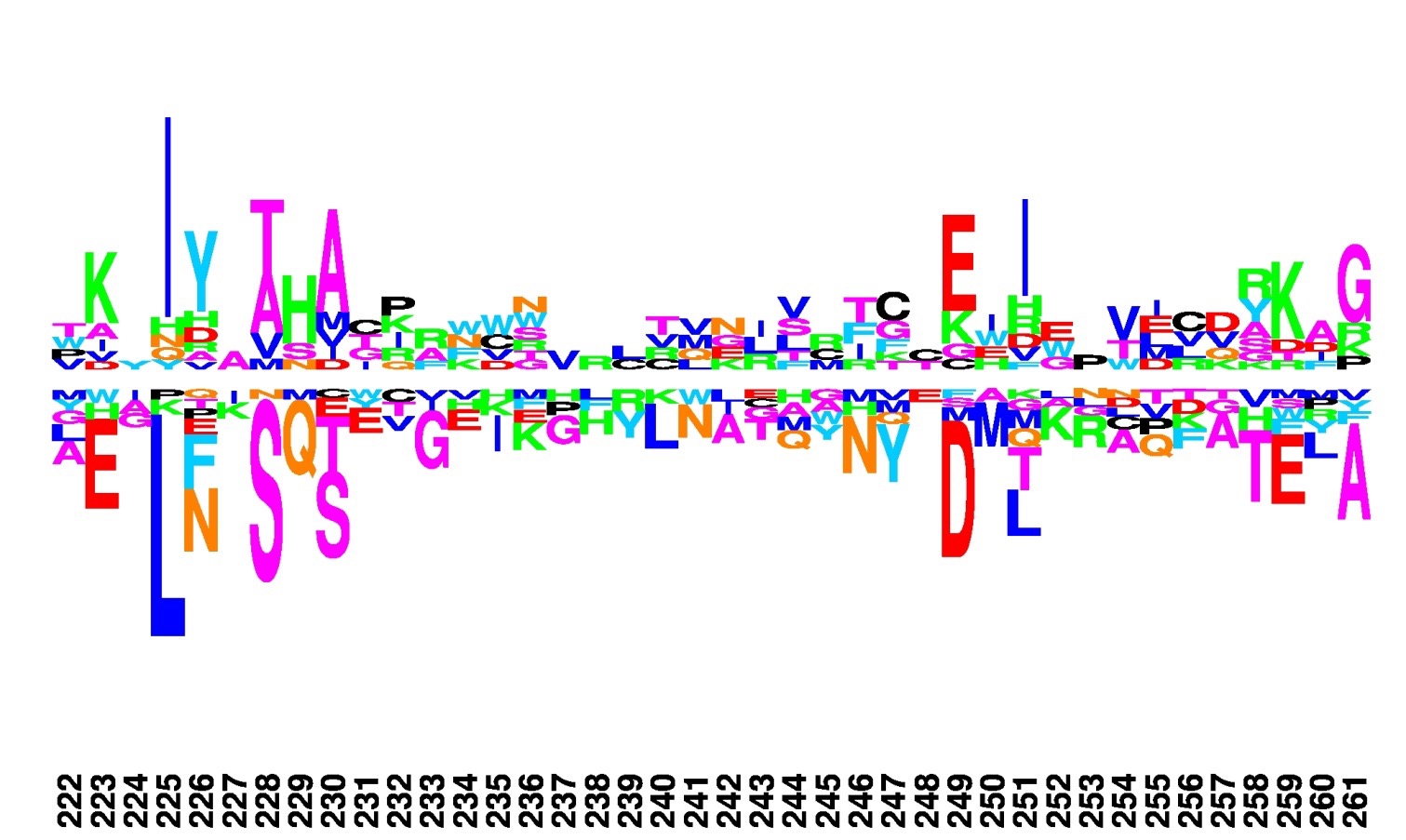 |
| 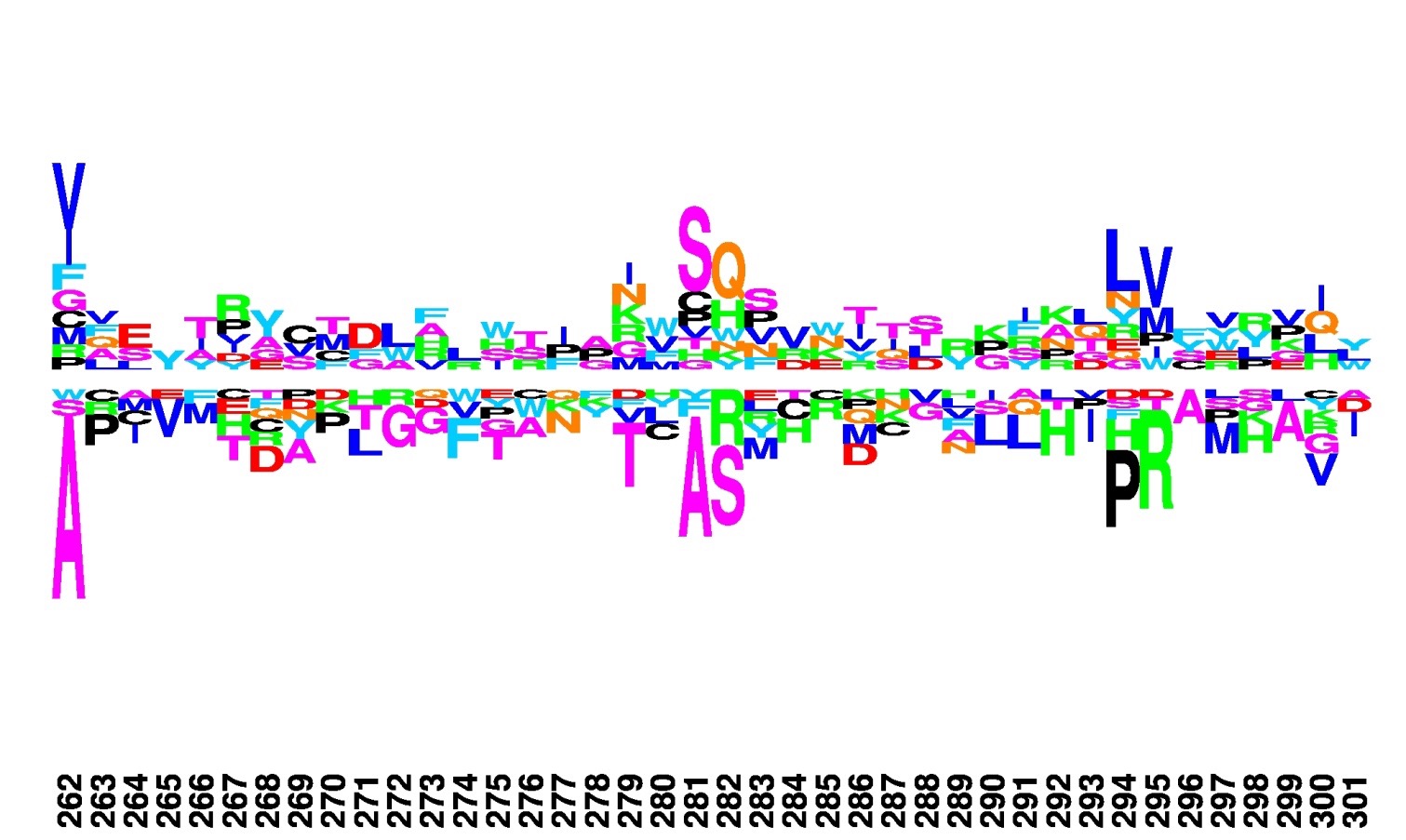 |
| 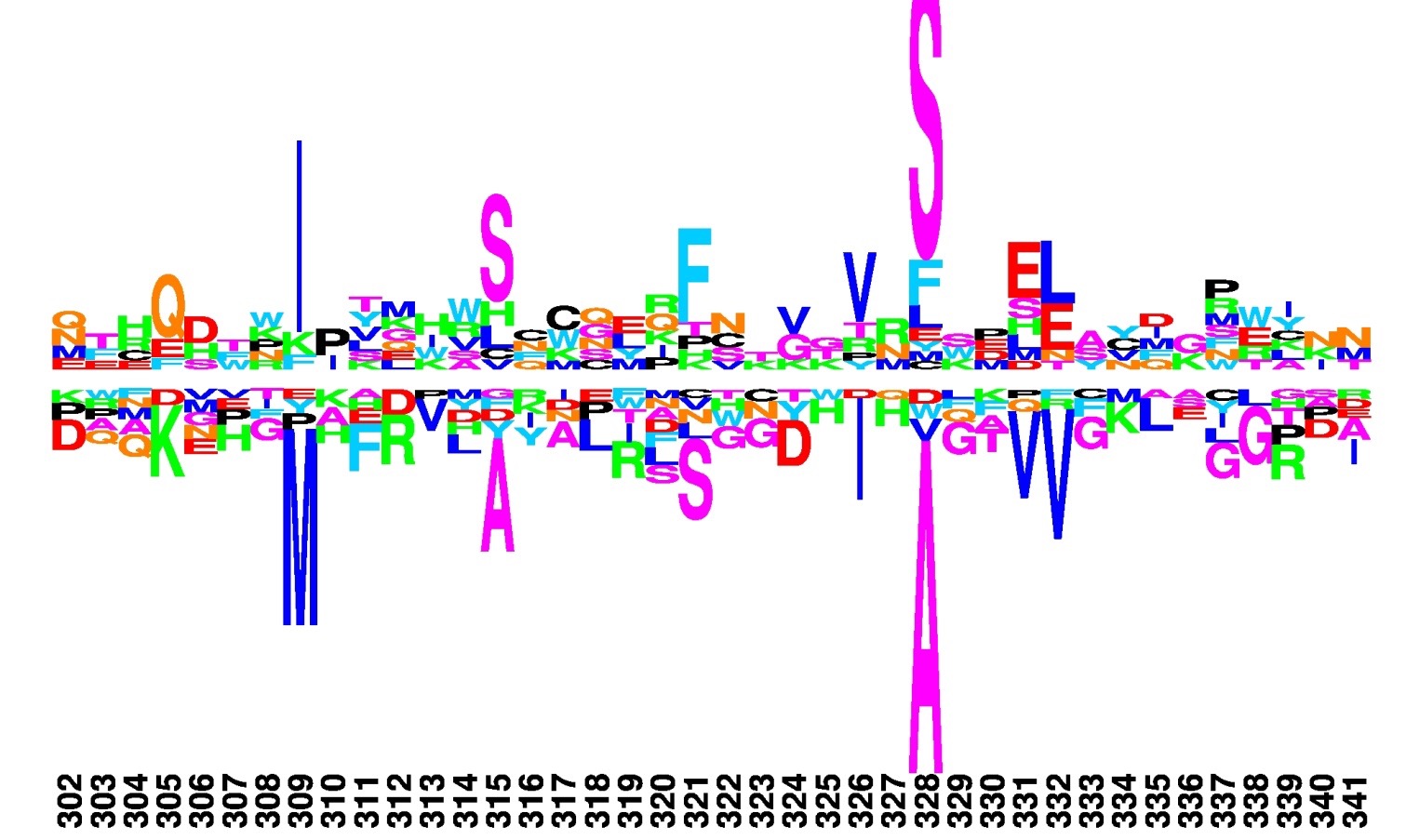 |
| 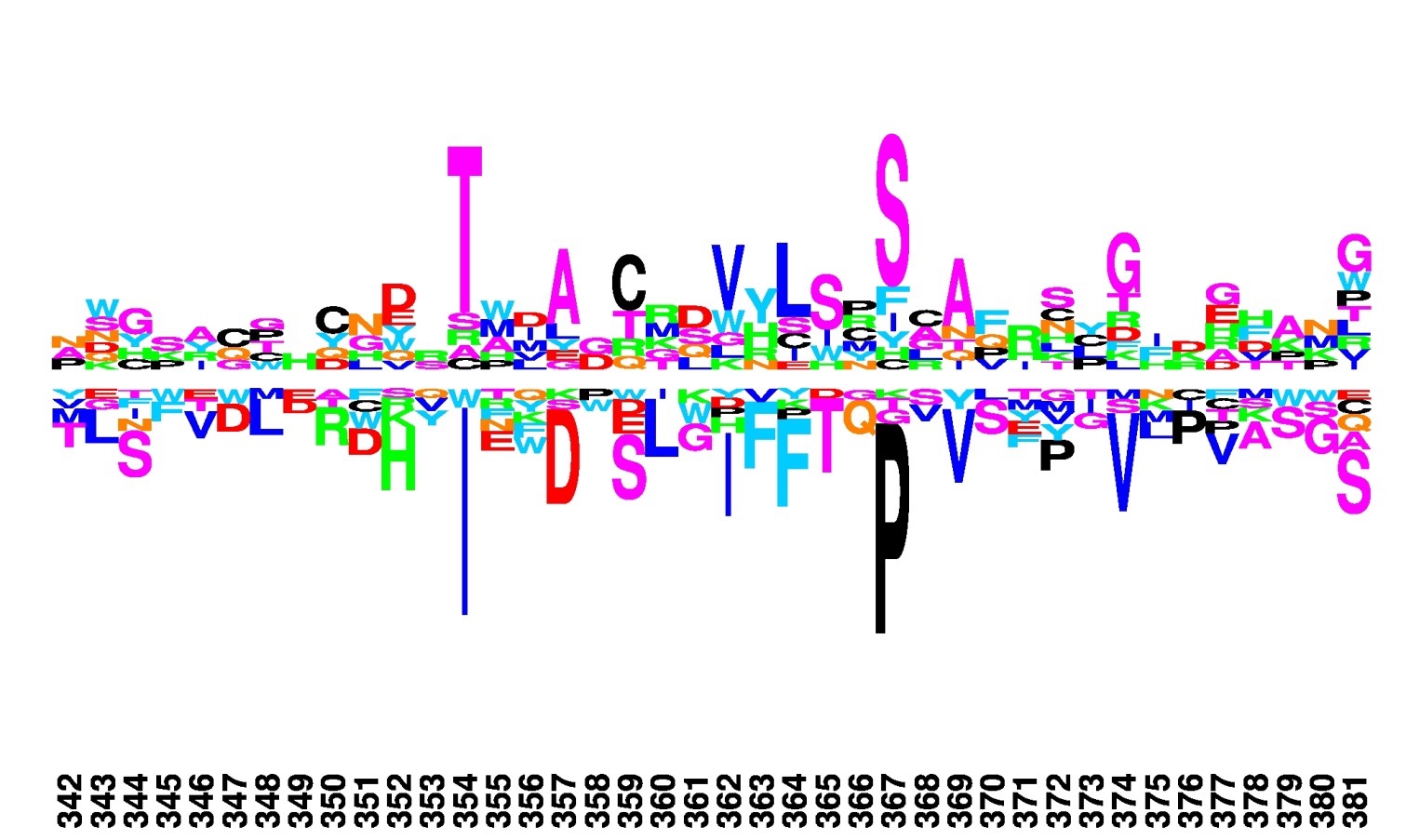 |
| 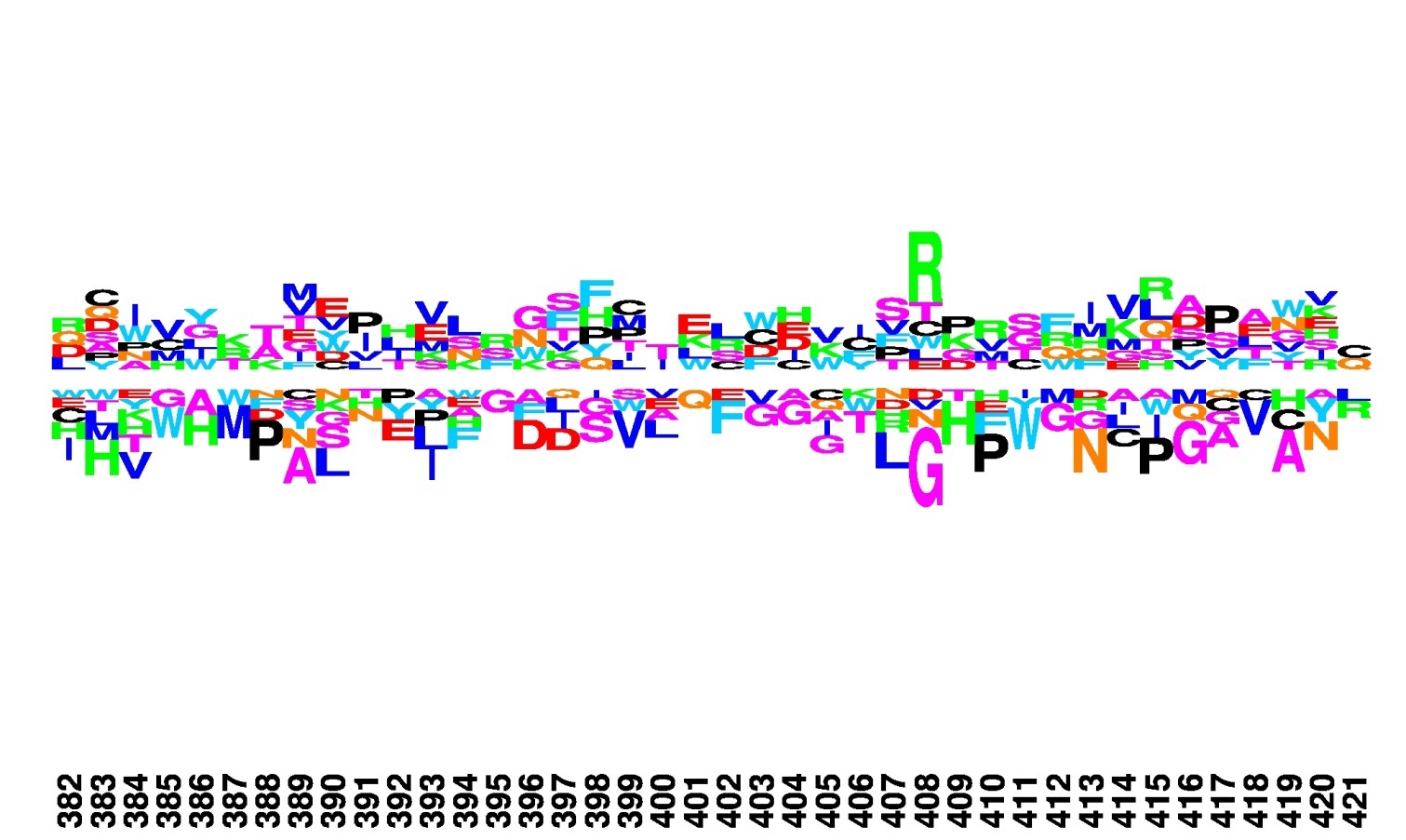 |
| 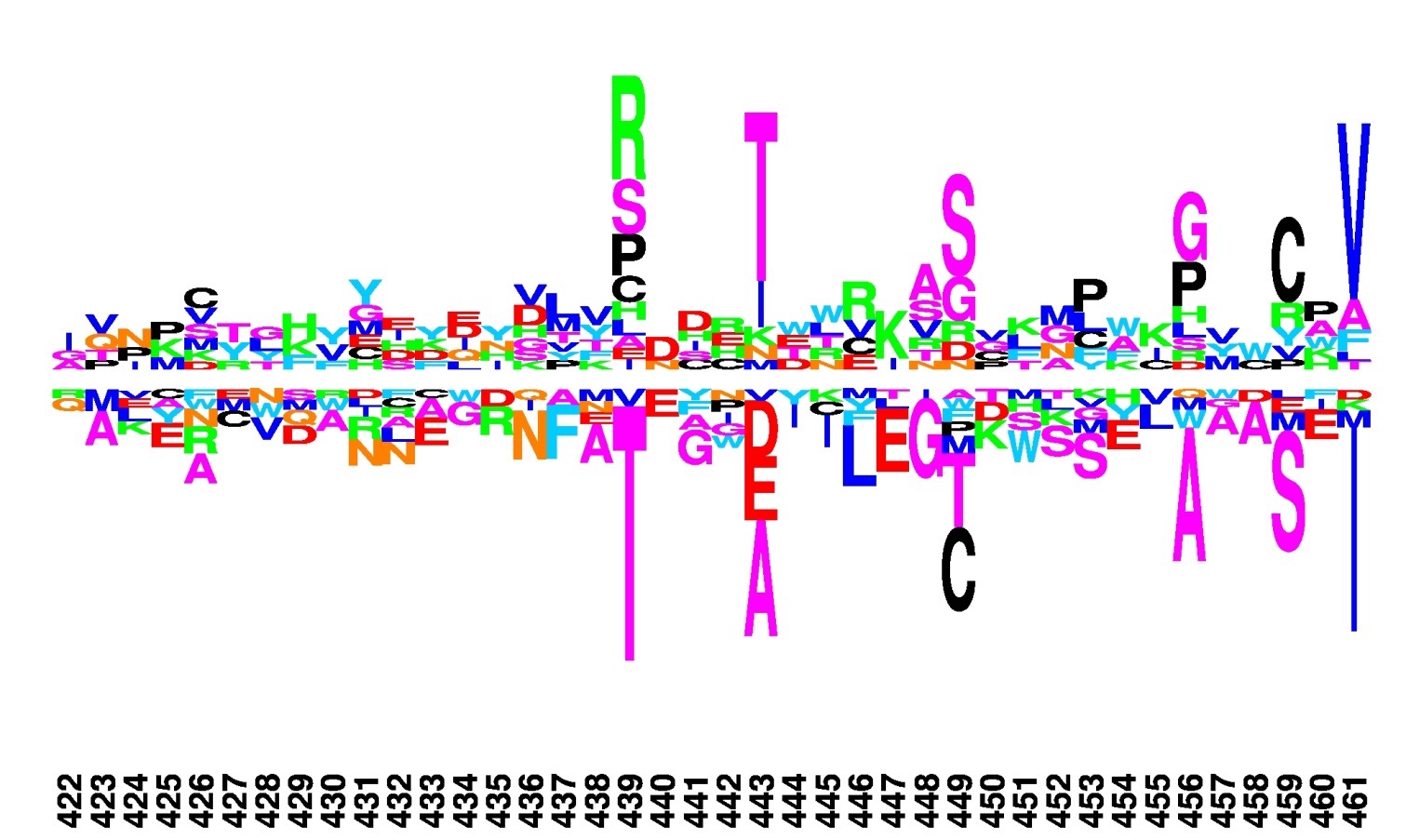 |
| 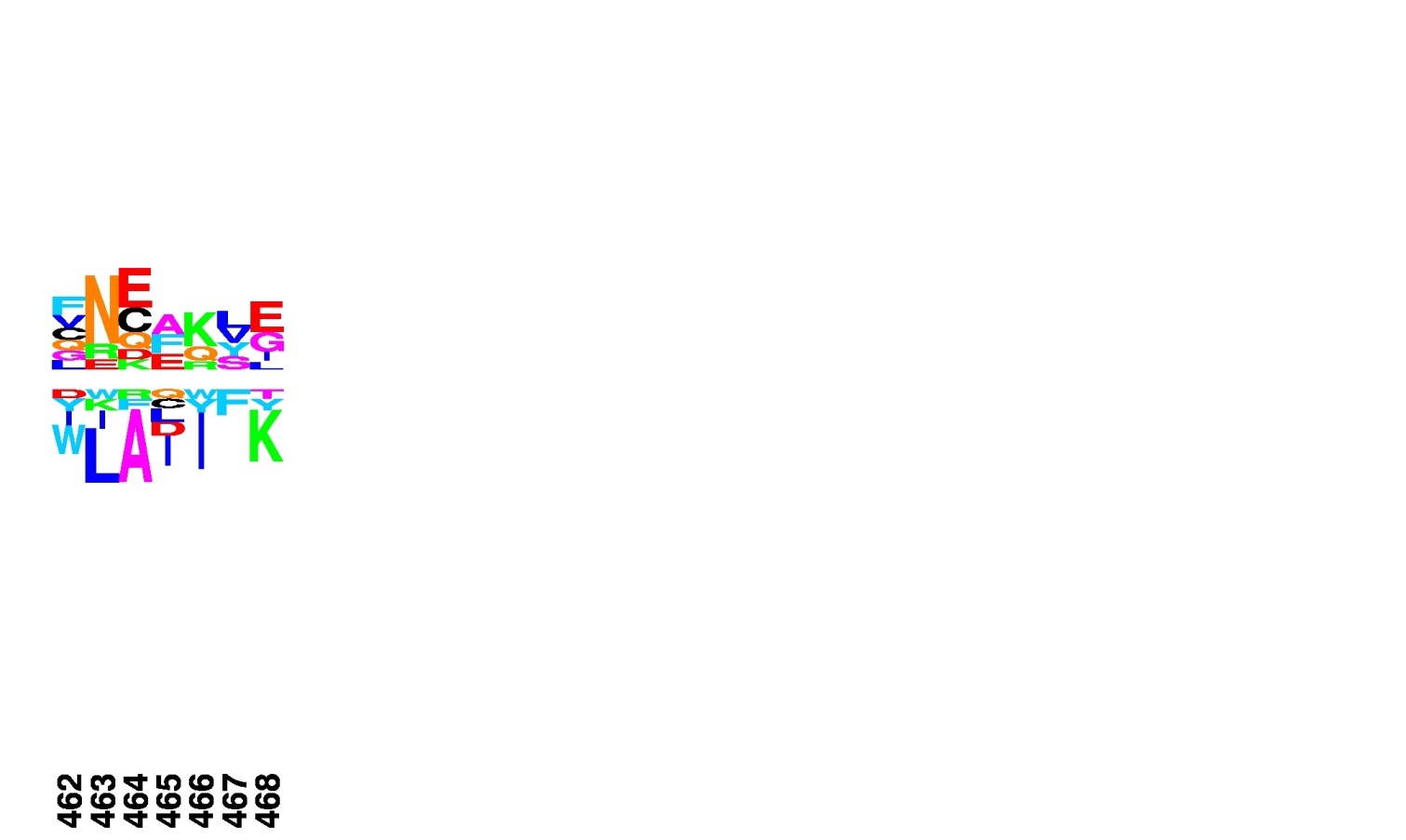 |

**Figure S1.** The C4 differential selection logo, for rbcL sequence in *Amaranthaceae* family. The first 21 amino acids are missing.
