# Supplementary material for: Molecular adaptation in Rubisco: Discriminating between convergent evolution and positive selection using mechanistic and classical codon models
Source: PLoS One. 2018 Feb 12;13(2):e0192697. doi: 10.1371/journal.pone.0192697 (PMC5809049; doi:10.1371/journal.pone.0192697)
Supplement: S1 Table — (DOCX) [file pone.0192697.s003.docx]

| **Species** | **Type of Photosynthesis** |
| --- | --- |
| Achyranthes_aspera | C3 |
| Acroglochin_chenopodioides | C3 |
| Aerva_javanica | C4 |
| Agriophyllum_squarrosum | C3 |
| Allenrolfea_occidentalis | C3 |
| Alternanthera_caracasana | C4 |
| Alternanthera_pungens | C4 |
| Alternanthera_repens | C4 |
| Amaranthus_blitum | C4 |
| Amaranthus_greggii | C4 |
| Amaranthus_hypochondriacus | C4 |
| Amaranthus_tricolor | C4 |
| Anabasis_aphylla | C4 |
| Anabasis_brevifolia | C4 |
| Anabasis_elatior | C4 |
| Anabasis_eriopoda | C4 |
| Anabasis_salsa | C4 |
| Anabasis_truncata | C4 |
| Anthochlamys_multinervis | C3 |
| Aphanisma_blitoides | C3 |
| Archiatriplex_nanpinensis | C3 |
| Arthrocnemum_macrostachyum | C3 |
| Atriplex_aucherii | C3 |
| Atriplex_australasica | C3 |
| Atriplex_centralasiatica | C4 |
| Atriplex_coriacea | C4 |
| Atriplex_glauca | C4 |
| Atriplex_halimus | C4 |
| Atriplex_lampa | C4 |
| Atriplex_lentiformis | C4 |
| Atriplex_parryi | C4 |
| Atriplex_patula | C3 |
| Atriplex_phyllostegia | C4 |
| Atriplex_powellii | C4 |
| Atriplex_rosea | C4 |
| Atriplex_serenana | C4 |
| Atriplex_spongiosa | C4 |
| Atriplex_undulata | C4 |
| Axyris_prostrata | C3 |
| Bassia_dasyphylla | C3 |
| Bassia_diffusa | C3 |
| Bassia_prostrata | C4 |
| Bassia_sedoides | C4 |
| Beta_nana | C3 |
| Beta_vulgaris | C3 |
| Bienertia_cycloptera | C4 |
| Blutaparon_vermiculare | C4 |
| Bosea_yervamora | C3 |
| Calicorema_capitata | C3 |
| Camphorosma_monspeliaca | C4 |
| Celosia_argentea | C3 |
| Celosia_trigyna | C3 |
| Ceratocarpus_arenarius | C3 |
| Chamissoa_altissima | C3 |
| Charpentiera_obovata | C3 |
| Charpentiera_ovata | C3 |
| Chenoleoides_tomentosa | C4 |
| Chenopodium_acuminatum | C3 |
| Chenopodium_album | C3 |
| Chenopodium_ambrosioides | C3 |
| Chenopodium_auricomum | C3 |
| Chenopodium_bonushenricus | C3 |
| Chenopodium_botrys | C3 |
| Chenopodium_coronopus | C3 |
| Chenopodium_cristatum | C3 |
| Chenopodium_desertorum | C3 |
| Chenopodium_foliosum | C3 |
| Chenopodium_frutescens | C3 |
| Chenopodium_murale | C3 |
| Chenopodium_sanctaeclarae | C3 |
| Climacoptera_brachiata | C4 |
| Climacoptera_lanata | C4 |
| Corispermum_filifolium | C3 |
| Cremnophyton_lanfrancoi | C3 |
| Cycloloma_atriplicifolium | C3 |
| Deeringia_amaranthoides | C3 |
| Dissocarpus_paradoxus | C3 |
| Dysphania_glomulifera | C3 |
| Einadia_nutans | C3 |
| Girgensohnia_oppositiflora | C4 |
| Gomphrena_elegans | C3 |
| Gomphrena_haageana | C4 |
| Gomphrena_serrata | C4 |
| Guilleminea_densa | C4 |
| Hablitzia_tamnoides | C3 |
| Halimione_pedunculata | C3 |
| Halimione_verrucifera | C3 |
| Halimocnemis_karelinii | C4 |
| Halimocnemis_villosa | C4 |
| Halocharis_hispida | C4 |
| Halogeton_arachnoideus | C4 |
| Halogeton_glomeratus | C4 |
| Halopeplis_amplexicaulis | C3 |
| Halosarcia_indica | C4 |
| Halostachys_belangeriana | C3 |
| Halothamnus_bottae | C4 |
| Haloxylon_ammodendron | C4 |
| Haloxylon_persicum | C4 |
| Haloxylon_tamariscifolium | C4 |
| Hebanthe_occidentalis | C3 |
| Hemichroa_diandra | C3 |
| Hermbstaedtia_glauca | C3 |
| Horaninovia_ulicina | C4 |
| Iljinia_regelii | C4 |
| Iresine_palmeri | C3 |
| Kalidium_caspicum | C3 |
| Kalidium_cuspidatum | C3 |
| Kalidium_foliatum | C3 |
| Kochia_americana | C3 |
| Kochia_densiflora | C4 |
| Krascheninnikovia_ceratoides | C3 |
| Maireana_brevifolia | C3 |
| Manochlamys_albicans | C3 |
| Microgynoecium_tibeticum | C3 |
| Micromonolepis_pusilla | C3 |
| Monolepis_nuttalliana | C3 |
| Nanophyton_erinaceum | C4 |
| Nitrophila_occidentalis | C3 |
| Noaea_mucronata | C4 |
| Nototrichium_humile | C3 |
| Ofaiston_monandrum | C4 |
| Oreobliton_thesioides | C3 |
| Pachycornia_triandra | C3 |
| Panderia_pilosa | C4 |
| Pandiaka_angustifolia | C3 |
| Patellifolia_patellaris | C3 |
| Petrosimonia_glaucescens | C4 |
| Petrosimonia_nigdeensis | C4 |
| Petrosimonia_sibirica | C4 |
| Petrosimonia_squarrosa | C4 |
| Polycnemum_perenne | C3 |
| Pseudoplantago_friesii | C3 |
| Ptilotus_manglesii | C3 |
| Pupalia_lappacea | C3 |
| Rhagodia_drummondi | C3 |
| Rhaphidophyton_regelii | C3 |
| Roycea_divaricata | C3 |
| Salicornia_dolichostachya | C3 |
| Salicornia_europaea | C3 |
| Salsola_affinis | C4 |
| Salsola_arbuscula | C4 |
| Salsola_arbusculiformis | C3 |
| Salsola_chinghaiensis | C4 |
| Salsola_collina | C4 |
| Salsola_dshungarica | C4 |
| Salsola_ferganica | C4 |
| Salsola_foliosa | C4 |
| Salsola_genistoides | C3 |
| Salsola_heptapotamica | C4 |
| Salsola_implicata | C4 |
| Salsola_kali | C4 |
| Salsola_komarovii | C4 |
| Salsola_laricifolia | C3 |
| Salsola_micranthera | C4 |
| Salsola_orientalis | C4 |
| Salsola_paulsenii | C4 |
| Salsola_pellucida | C4 |
| Salsola_praecox | C4 |
| Salsola_rosacea | C4 |
| Salsola_ruthenica | C4 |
| Salsola_sukaczevii | C4 |
| Salsola_vermiculata | C4 |
| Salsola_zaidamica | C4 |
| Sarcocornia_utahensis | C3 |
| Sclerolaena_obliquicuspis | C3 |
| Sclerostegia_moniliformis | C3 |
| Sericostachys_scandens | C3 |
| Spinacia_oleracea | C3 |
| Suaeda_altissima | C4 |
| Suaeda_crassifolia | C3 |
| Suaeda_maritima | C3 |
| Suaeda_microphylla | C4 |
| Suaeda_physophora | C3 |
| Suckleya_suckleyana | C3 |
| Sympegma_regelii | C3 |
| Tecticornia_australasica | C3 |
| Tecticornia_disarticulata | C3 |
| Teloxys_aristata | C3 |
| Tidestromia_lanuginosa | C4 |
